# Single gene microdeletions and microduplication of 3p26.3 in three unrelated families: *CNTN6* as a new candidate gene for intellectual disability

**DOI:** 10.1186/s13039-014-0097-0

**Published:** 2014-12-31

**Authors:** Anna A Kashevarova, Lyudmila P Nazarenko, Soren Schultz-Pedersen, Nikolay A Skryabin, Olga A Salyukova, Nataliya N Chechetkina, Ekaterina N Tolmacheva, Aleksey A Rudko, Pamela Magini, Claudio Graziano, Giovanni Romeo, Shelagh Joss, Zeynep Tümer, Igor N Lebedev

**Affiliations:** Laboratory of Cytogenetics, Institute of Medical Genetics, 10 Nab. Ushaiki, 634050 Tomsk, Russia; Laboratory of Hereditary Pathology, Institute of Medical Genetics, Tomsk, Russia; Department of Medical Genetics, Siberian State Medical University, Tomsk, Russia; Private Pediatric Clinics “Bornelaegen Aarhus”, Aarhus, Denmark; Laboratory of Human Ontogenetics, Tomsk State University, Tomsk, Russia; Genetic Clinic, Institute of Medical Genetics, Tomsk, Russia; Unit of Medical Genetics, Department of Gynecology, Obstetrics and Pediatrics, University of Bologna, Bologna, Italy; Department of Clinical Genetics, Level 2, Laboratory Medicine Building, Southern General Hospital, Glasgow, G51 4TF Scotland UK; Applied Human Molecular Genetics, Kennedy Center, Department of Clinical Genetics, Copenhagen University Hospital, Rigshospitalet, Copenhagen Denmark

**Keywords:** Intellectual disability, 3p26.3 microdeletion, 3p26.3 microduplication, *CNTN6*, Reciprocal microdeletions/microduplications

## Abstract

**Background:**

Detection of submicroscopic chromosomal alterations in patients with a idiopathic intellectual disability (ID) allows significant improvement in delineation of the regions of the genome that are associated with brain development and function. However, these chromosomal regions usually contain several protein-coding genes and regulatory elements, complicating the understanding of genotype-phenotype correlations. We report two siblings with ID and an unrelated patient with atypical autism who had 3p26.3 microdeletions and one intellectually disabled patient with a 3p26.3 microduplication encompassing only the *CNTN6* gene.

**Results:**

Two 295.1-kb microdeletions and one 766.1-kb microduplication of 3p26.3 involving a single gene, *CNTN6*, were identified with an Agilent 60K array. Another 271.9-kb microdeletion of 3p26.3 was detected using an Affymetrix CytoScan HD chromosome microarray platform. The *CHL1* and *CNTN4* genes, although adjacent to the *CNTN6* gene, were not affected in either of these patients.

**Conclusions:**

The protein encoded by *CNTN6* is a member of the immunoglobulin superfamily and functions as a cell adhesion molecule that is involved in the formation of axon connections in the developing nervous system. Our results indicate that *CNTN6* may be a candidate gene for ID.

**Electronic supplementary material:**

The online version of this article (doi:10.1186/s13039-014-0097-0) contains supplementary material, which is available to authorized users.

## Background

Most chromosomal microdeletions and microduplications encompass several genes with various functions, creating a challenge in understanding genotype-phenotype correlations. Occasionally, however, these mutations may involve a single gene or a portion of a gene, blurring the border between chromosomal and monogenic diseases [1,2]. Such cases are of special interest in analyzing the clinical effects of dosage variations of a single gene due to chromosomal microdeletions or microduplications, which may form “genomic sister disorders” [3-5]. To date, more than 56 of these syndromes have been described, and the list of genomic sister disorders is constantly being updated [6].

The rare and incompletely understood 3p distal deletion syndrome is characterized by growth retardation, developmental delay, ID, microcephaly, ptosis and dysmorphisms. Polydactyly, renal abnormalities, cleft palate, congenital heart defects (particularly atrioventricular septal defects), preauricular pits, sacral dimple and gastrointestinal anomalies are variable features of this syndrome [Online Mendelian Inheritance in Man (OMIM) 613792] [7]. Patient phenotypes can vary from normal to severe. These deletions usually include several genes. The minimal deletion region is thought to be 1.5 Mb in size and to include the *CRBN* and *CNTN4* genes [8]. However, an additional gene, *CHL1*, is also considered to be critical for mental development because it is highly expressed in the brain [9]. There are no common breakpoints in this genomic region, and deletions generally occur *de novo*; however, a few familial cases have been described [9,10]. *CNTN6* is located between *CHL1* and *CNTN4.* Because it is expressed (among other organs) in the brain it may contribute to the development of ID. Therefore, analysis of microdeletions and microduplications affecting this gene may be of particular relevance for the distal 3p deletion syndrome.

In contrast with deletions, isolated duplications of the terminal region of the 3p have been less frequently reported. Two patients with a 3p26.3 microduplication that fully or partially encompasses *CHL1* [11,12] have been reported so far. The first patient, who had ID and epilepsy, carried a single *CHL1* gene duplication [11], while the duplication in the second reported patient encompassed the *CNTN6* gene along with *CHL1*. This patient presented with motor and speech delays and some autistic features [12]. In a large cohort of patients with autism spectrum disorder (ASD), a single inherited duplication of exons 1–4 and two inherited deletions of exons 1–2 and exon 5 in the *CNTN6* gene were found [13]. An isolated *de novo* duplication of the first two *CNTN6* exons in a patient with an autistic disorder was also reported by van Daalen and colleagues [14]. The authors considered the duplication to be sufficient to cause ASD in the affected proband. However, an isolated duplication or deletion of *CNTN6* has never been described in patients with an ID.

We report for the first time two siblings with 295.1-kb microdeletions, an unrelated patient with a 271.9-kb microdeletion and an additional patient with a 766.1-kb microduplication of 3p26.3, all of which encompass a single gene, *CNTN6*. The patients with the 295.1-kb microdeletions and 766.1-kb microduplication presented with developmental delay, ID, speech and language delays, an abnormal skull shape and facial dysmorphism. The patient with the 271.9-kb microdeletion had mild motor development delay, ID, atypical autism, and speech delay.

## Results

### Family F

**Figure 1:**
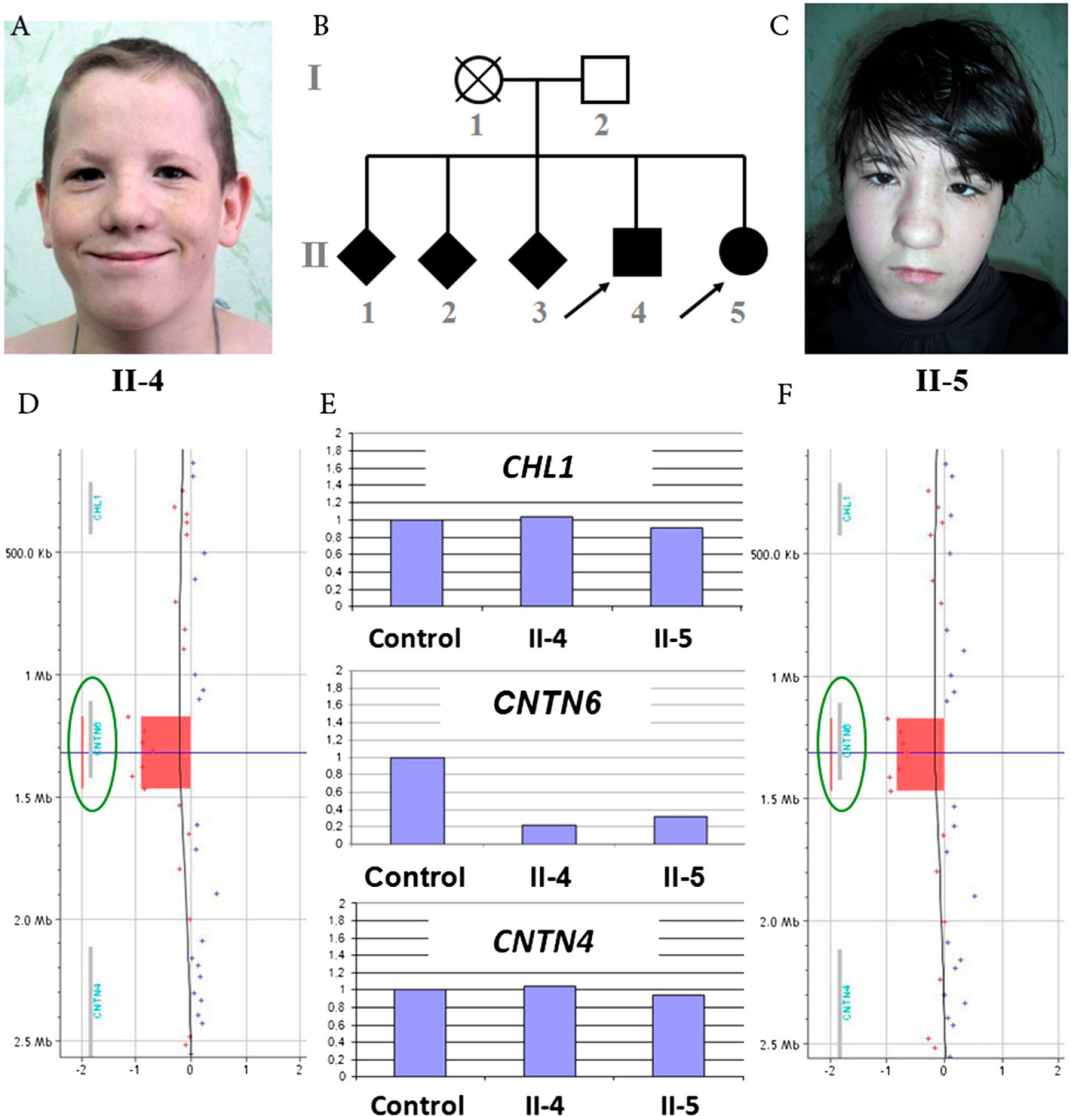
**Analysis of the siblings from family F.** **(** **A** **)** The male patient F (note the tower skull, frontal bossing, antimongoloid slant, epicanthus, wide nasal bridge, large nose, anteverted nostrils, and low-set ears). **(** **B** **)** The pedigree plot for family F; the solid square and circle represent the affected siblings. **(** **C** **)** The female patient F (note the microcephaly, long face, epicanthus, wide nasal bridge, and short philtrum). **(** **D** **)** The *CNTN6* deletion in the male patient F. **(** **E** **)** Real-time PCR analysis of *CHL1, CNTN6*, and *CNTN4*, which revealed two copies of *CHL1*, a deletion of *CNTN6*, and two copies of *CNTN4* in both patients, respectively. **(** **F** **)** The *CNTN6* deletion in the female patient F.

The female patient from family F (Figure 1) was born at the 33^rd^ week of gestation. Her weight was 2,000 g (3^rd^ centile), and her height was 48 cm (10^th^ centile). Her Apgar score was 7. From 4 years of age, a psychiatrist had determined that the girl had mild ID and behavioral problems. Logopedic examination revealed dyslexia and dysgraphia. The girl had moderate hyperopia and severe amblyopia. Ultrasound examination revealed thyroid hypoplasia, bilateral pyelectasia, and right nephroptosis.

At 14 years of age, her height was 145 cm (3^rd^ centile), her weight was 40 kg (3^rd^ centile), and head circumference was 48 cm (3^rd^ centile). A clinical examination revealed microcephaly, a long face, epicanthus, wide nasal bridge, small ears, short philtrum, crowding of teeth, peg-shaped lateral incisors, bilateral clinodactyly of the fifth finger, bilateral sandal gap, and X-shaped legs.

This patient had clear consciousness and was normally calm, friendly, and shy; however, she was occasionally hysterical and aggressive. She readily answered questions, but her answers were abrupt and primitive. Occasionally, she did not hear the questions or provided an irrelevant answer. Her emotional reactions were generally monotonous and primitive. Her general knowledge was limited to her everyday interests; she could not think abstractly. In addition, she became easily tired and weakened. Her IQ was 54.

Her brother (Figure 1) was born at the 33^rd^ week of gestation. His weight was 1,550 g (3^rd^ centile), and his height was 44 cm (3^rd^ centile). His Apgar score was 6–7. During pregnancy his mother had syphilis, pyelonephritis, and bronchitis. The child was born with umbilical cord entanglement. From the anamnesis, after discharge from the maternity hospital, he was treated for syphilis. He displayed physical and psychomotor developmental delays. At 6 years, he was transferred from a regional orphanage. A radiograph of the wrist joints at 14 years of age revealed a bone age of 6–7 years. The neurologist diagnosed an absence epilepsy, dysarthria, and short stature. Ultrasound examination revealed hypoplastic left lobe of thyroid gland and testicular hypoplasia. This boy exhibited exhaustible attention, reduced short-term and long-term memory, and low adaptive ability. His IQ was 49.

The last evaluation of the patient by a clinical geneticist was at the age of 15. His height at this time was 140 cm (3^rd^ centile), his weight was 34 kg (3^rd^ centile), and his head circumference was 51 cm (3^rd^ centile). Examination revealed towered skull, frontal bossing, antimongoloid slant, epicanthus, wide nasal bridge, large nose, anteverted nostrils, low-set ears, gothic palate, irregular growth of the teeth, bilateral clinodactyly of fifth finger, bilateral sandal gap, scoliosis, non-malignant pigmented nevus in the right inguinal region, and hypertrichosis.

The copy number variations (CNVs) discovered via array comparative genomic hybridization (CGH) using an Agilent 60K platform showed small deletion within 3p26.3 in both siblings (Table 1). The centromeric breakpoint of the deletion was located within the intergenic sequence between *CNTN6* and *CNTN4*, and the telomeric breakpoint was located in the 2^nd^ intron of *CNTN6*. The deletion was confirmed with real-time PCR using two primer pairs that were specific to *CNTN6* (Figure 1). The integrities of *CNTN4*, which is located proximal to the centromere, and *CHL1*, which is located distal to *CNTN6* toward the telomere, were also confirmed via real-time PCR (Figure 1). These siblings were orphans; therefore, it was impossible to determine the parental origin of the deletion.

**Table 1:** **CNVs in patients F, K, and N who had ID, and in the relatives of patient K**

**Patient**	**Array CGH results (according to ISCN (2013)) [** 15 **]**
Female F	arr[hg18]**3p26.3(1,172,623-1,467,721) × 1**, 10q11.22(45,478,103-47,125,152) × 3
Male F	arr[hg18]**3p26.3(1,172,623-1,467,721) × 1**
Female N	arr[hg18]**3p26.3(1,403,385 -1,675,322) × 1 pat**
Male K	arr[hg18]**3p26.3(701,645-1,467,721) × 3 pat**, 6p22.1p21.33(29870108–29,981,971) × 3, 21q22.11(32,379,083-32,543,206) × 3
Father K	arr[hg18]2p11.2(89,649,638-89,871,328) × 1, 2q13(110,253,270-110,327,559) × 1, **3p26.3(608,449-1,533,564) × 3 mat**
Grandmother K	arr[hg18]**3p26.3(701,645-1,533,564) × 3**, 14q11.2(21,457,258-21,936,635) × 1

Footnote. Indexed CNVs are shown in bold; polymorphisms are in Roman font.

### Family N

The pregnancy and birth of that girl were uneventful. Her birth weight was 3220 g (50^th^ centile), and her birth height was 52 cm (75^th^ centile). Her motor development was slightly delayed. She started walking at the age of 1 year and 4 months, but her gait was unsteady for a long time. She had hypermobile joints, which were also present in the father and the paternal grandmother according to the parents.

She was administered the motor and perceptual development test at the age of 2 years and 2 months, and the results suggested that she had problems with tasks requiring coordination, strength, and balance. Her cognitive functions were apparently age-matched, but at 3½ years of age, her concentration ability was clearly low. Speech development was also slightly delayed.

This patient had support during kindergarten and attended a special school class. At 13 years of age, her IQ was 40. The same year, neurological examination revealed a tall and well-built girl with no dysmorphic features. She showed some anxiety and needed a clear explanation before performing tests. She had obvious tactile shyness and low perception ability. Her balance was good, but she showed low stamina during the balance performance test. Her overall contact was impaired. She has been recently diagnosed as having atypical autism.

Her somatic record was good with the exception of intermediate constipation.

Magnetic resonance imaging (MRI) scans of the brain performed at 6 years of age and repeated at 14 years of age were normal.

**Figure 2:**
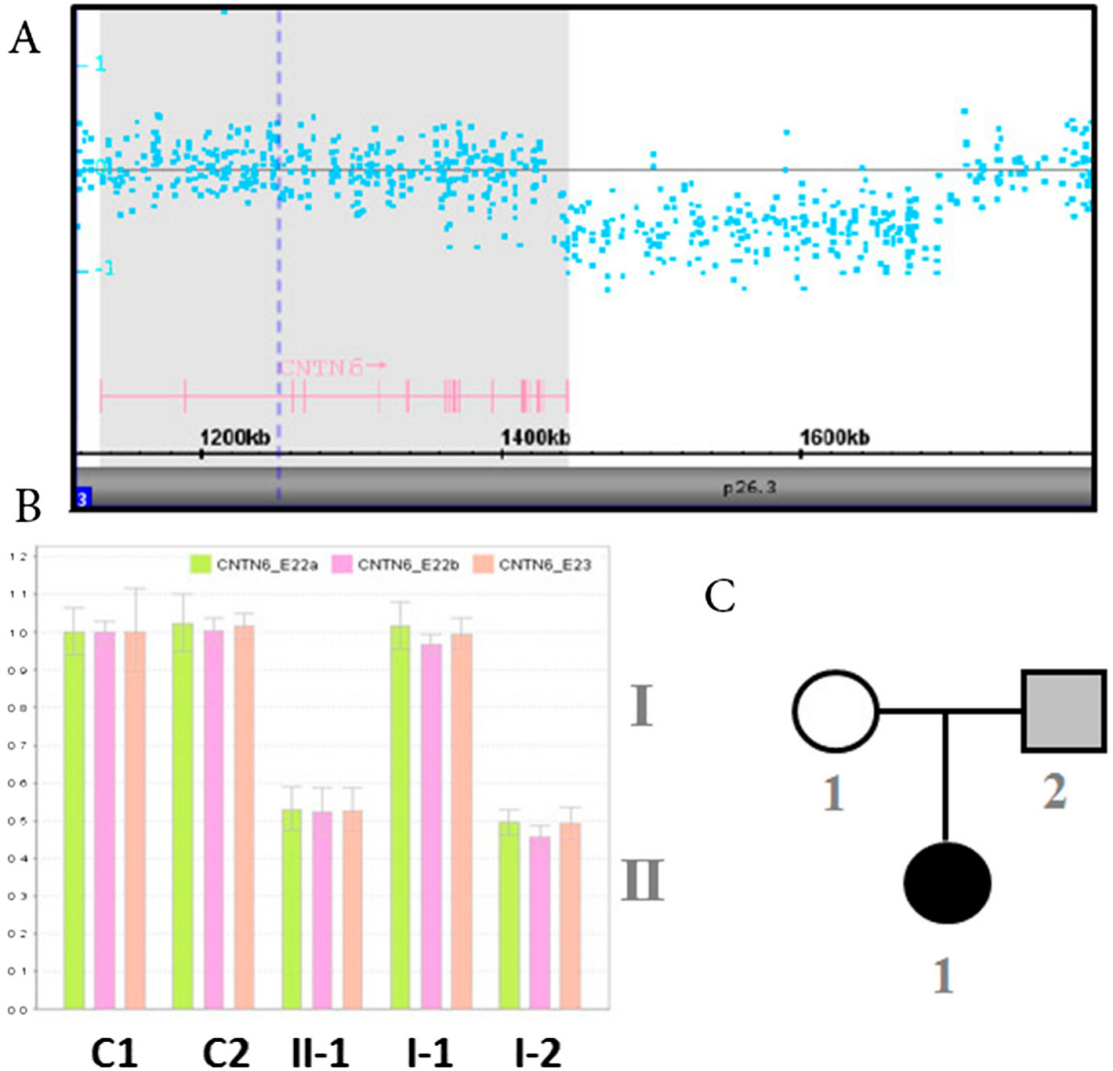
**Analysis of family N.** **(** **A** **)** The *CNTN6* deletion in patient N. **(** **B** **)** Real-time PCR analysis of *CNTN6*: C1 - female control, C2 - male control, II-1 - patient N, I-1 - mother, I-2 - father; CNTN6_E22a and CNTN6_E22b - probes for exon 22, CNTN6_E23 - probe for exon 23. **(** **C** **)** The pedigree plot for family N; the dark, solid circle represents the affected patient N, and the gray, solid square represents father N.

The microdeletion of 3p26.3 discovered via array CGH using an Affymetrix CytoScan HD chromosome microarray platform to assess patient N is shown in Table 1. The del3p26.3 centromeric breakpoint was located within the intergenic sequence between *CNTN6* and *CNTN4*, and the telomeric breakpoint was located in the 20^th^ intron of *CNTN6*. The deletion was confirmed with real-time PCR using primers that were specific to *CNTN6*, and it was determined to be inherited from the apparently healthy father (Figure 2).

### Family K

**Figure 3:**
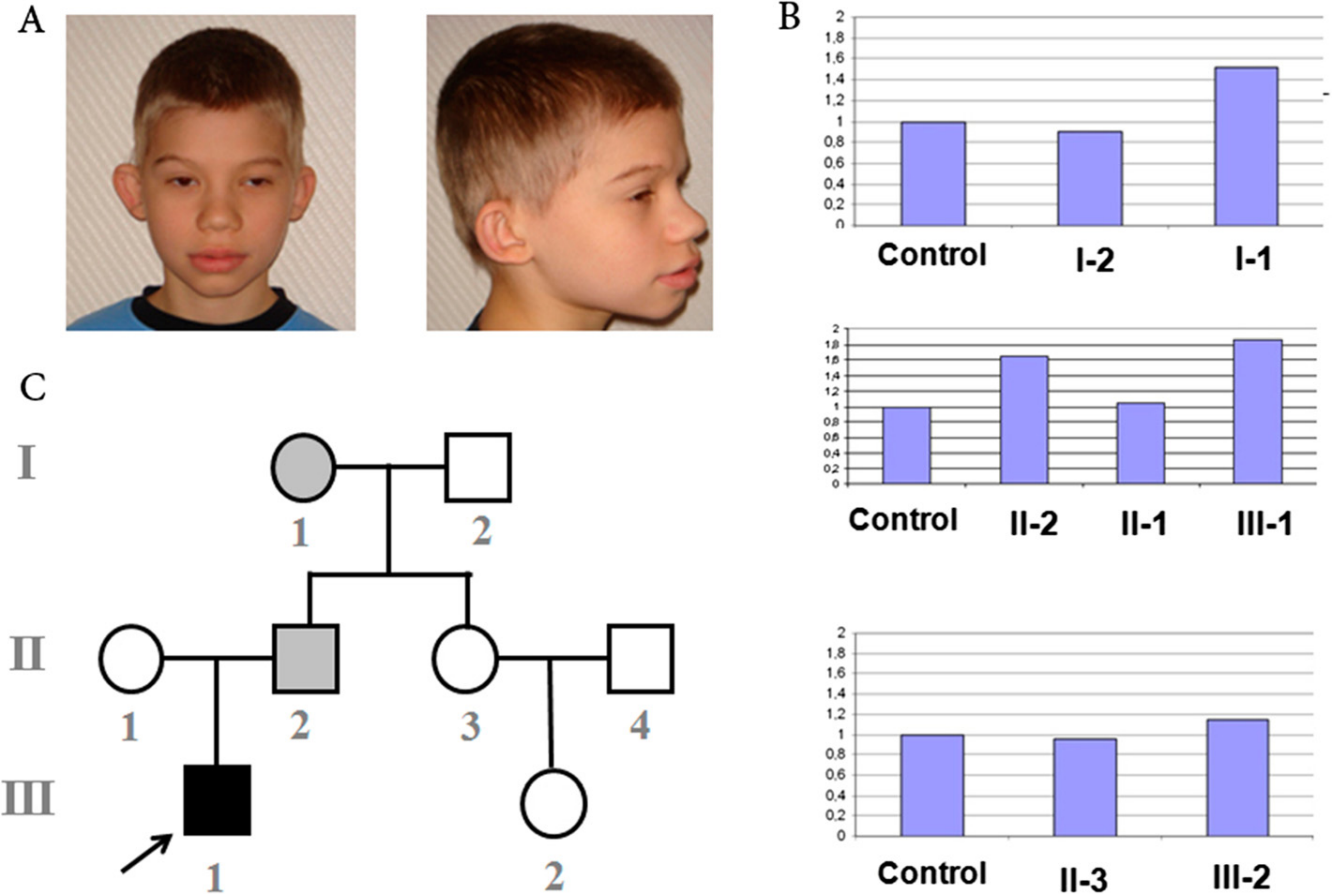
**Analysis of family K.** **(** **A** **)** Patient K (note the dolichocephalic skull, upslanting palpebral fissures, convergent strabismus, epicanthus, wide nasal bridge, small nose, low-set protruding ears with malformed helices, macrostomia, micrognathia, short philtrum, and short neck). **(** **B** **)** Real-time PCR analysis of *CNTN6*; upper panel, the duplication of *CNTN6* in grandmother K (I-1); middle panel, the duplication of *CNTN6* in father K (II-2) and proband K (III-1); and lower panel, the two copies of *CNTN6* in patient’s K aunt (II-3) and her daughter (III-2). **(** **C** **)** The pedigree plot for family K; the dark, solid square represents the affected patient K, and the gray, solid square and circle represent father K and grandmother K, respectively.

The male patient from family K (Figure 3) was born at the 42^nd^ week of gestation. His weight was 3,300 g (50^th^ centile), his height was 53 cm (90^th^ centile), and his head circumference was 35 cm (50^th^ centile). His Apgar score was 7. He displayed physical and psychomotor developmental delays (sitting at 8 months, walking at 1 year and 6 months, first words at 2 years, and phrases at 5 years). This patient was hyperreactive until 1 year of age.

At 9 years of age, his height was 131 cm (50^th^ centile), his weight was 25 kg (50^th^ centile), and his head circumference was 50 cm (3^rd^ centile). A clinical examination at this time revealed a dolichocephalic skull, unusual and coarse hair, a low posterior hair line, direct, high and wide set eyebrows, upslanting palpebral fissures, convergent strabismus, epicanthus, a wide nasal bridge, small nose, low-set protruding ears with malformed helices, macrostomia, micrognathia, short philtrum, high palate, short neck, hypermobility of the small and large joints, hypotonia, scoliosis, cryptorchidism, and hypospadias. His fine motor skills were impaired. He had speech delay, dysarthria, hyperkineses and attention-deficit/hyperactivity disorder. His IQ was 47. Cardiologic examination revealed an additional chord in the left ventricle. Computer tomography of the brain did not reveal organic lesions.

**Figure 4:**
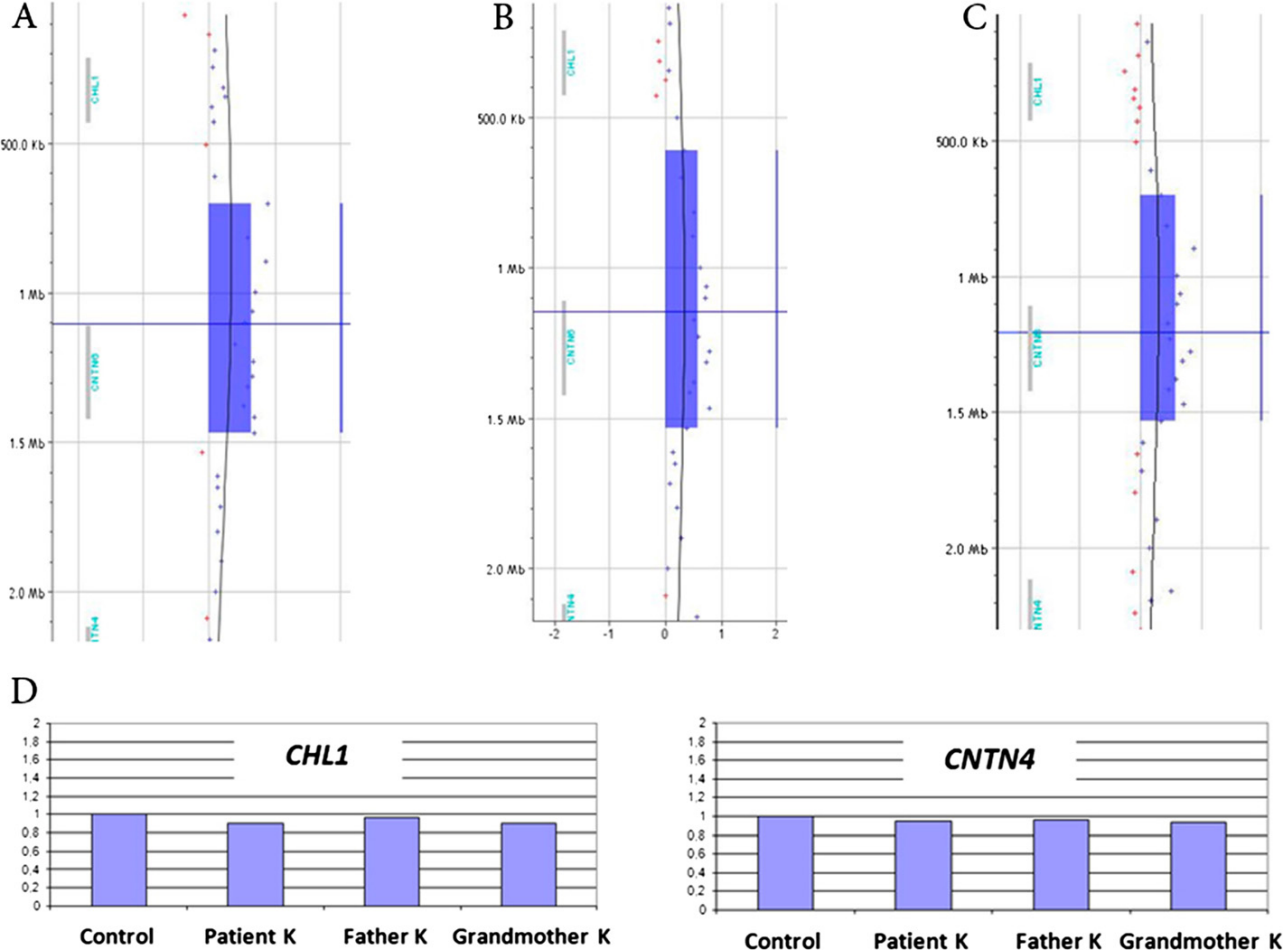
**Array CGH and real-time PCR analyses of family K.** **(** **A** **)** The *CNTN6* duplication in patient K, father K and grandmother K. **(** **B** **)** The *CNTN6* duplication in father K. **(** **C** **)** The *CNTN6* duplication in grandmother K. **(** **D** **)** Left to right: two copies of *CHL1* and two copies of *CNTN4* in patient F, father F and grandmother F.

Array CGH using an Agilent 60K platform revealed a small duplication of 3p26.3 in patient K and his relatives (Table 1, Figure 3). The centromeric breakpoint of this rearrangement in patient K was located within the intergenic region between *CNTN6* and *CNTN4*, and the telomeric breakpoint was located within the intergenic region between *CNTN6* and *CHL1*; therefore, the entire *CNTN6* was duplicated. This duplication was confirmed with real-time PCR using two primer pairs that were specific to *CNTN6*. Real-time PCR analysis revealed that the microduplication was inherited from an apparently healthy father who had inherited it from his apparently healthy mother (Figure 3). The integrity of *CNTN4* and *CHL1*, which are located on either side of *CNTN6*, was confirmed with real-time PCR in all cases (Figure 4). No pathogenic CNVs were discovered in the patient’s aunt and her daughter (Figure 3).

## Discussion

Single-gene chromosomal microdeletions and microduplications are unique and very important for evaluating the clinical effects of dosage alterations of the involved gene. In this report, we presented two sibs with ID and a 295.1-kb 3p26.3 microdeletion, a girl with ID, atypical autism, and a 271.9-kb 3p26.3 microdeletion inherited from an apparently healthy father, and a boy with ID and a 766.1-kb 3p26.3 microduplication inherited from an apparently healthy father (925.1 kb) and grandmother (831.9 kb). In all of the affected individuals, the aberration spanned less than one megabase and included only one Refseq gene, *CNTN6.* In the microdeletion found in the siblings, exons 3–23 were absent, and the microdeletion of patient N led to the absence of exons 21–23, while in family K, the entire gene was duplicated.

The *CNTN6* gene is located in the region which includes several more genes (*CNTN4, CRBN, CHL1*) involved in the clinical picture of the distal 3p deletion syndrome [16]. Therefore, to delineate the pathogenicity of alterations in *CNTN6*, it is important to compare the clinical features associated with the isolated single-gene microdeletion or microduplication and the contiguous gene syndrome to determine common and unique symptoms.

The siblings from family F had ID and certain features of the distal 3p deletion syndrome, including low birth weight, psychomotor and growth retardation, and microcephaly (Additional file 1: Table S1). The male also had downslanting palpebral fissures, and the female had renal anomalies. Patient N had mild motor development delay during infancy and hypotonia associated with the 3p deletion syndrome. She was also intellectually disabled. Patient K, who had a microduplication of 3p26.3, also demonstrated ID as well as psychomotor and growth retardation, microcephaly, which have been described also in patients with distal 3p deletion syndrome. The classification of clinical phenotypes in reciprocal microdeletion and microduplication syndromes introduced by Golzio and Katsanis suggests that the phenotypes of patients with a microdeletion and those of patients with a reciprocal microduplication may be identical in some cases [17].

In all of our four patients, the following symptoms were identified independent of the chromosomal aberration type: ID and psychomotor and growth retardation (mild motor development delay in patient N). In the siblings from family F and in the patient K, microcephaly and additional dysmorphic features (epicanthus, wide nasal bridge) were observed. A high palate, low-set ears, and moderate intracranial hypertension were detected only in the male from family F with the microdeletion and the male with the microduplication, while a short philtrum was present in the female from family F with the microdeletion and the male with the microduplication. The birth weights of both siblings with the microdeletion were much lower than those in the third patient with the microdeletion and the patient with the microduplication (3^rd^ centile vs. 50^th^ centile, respectively). Furthermore, in both patients from family F with del3p26.3, in addition to the known features of 3p deletion syndrome, teeth-crowding, peg-shaped lateral incisors, scoliosis, bilateral sandal gap, bilateral fifth finger clinodactyly, and thyroid gland hypoplasia were identified. Hypermobility of the joints was observed in the patient with the microduplication from family N, which was also found in her father and paternal grandmother.

**Figure 5:**
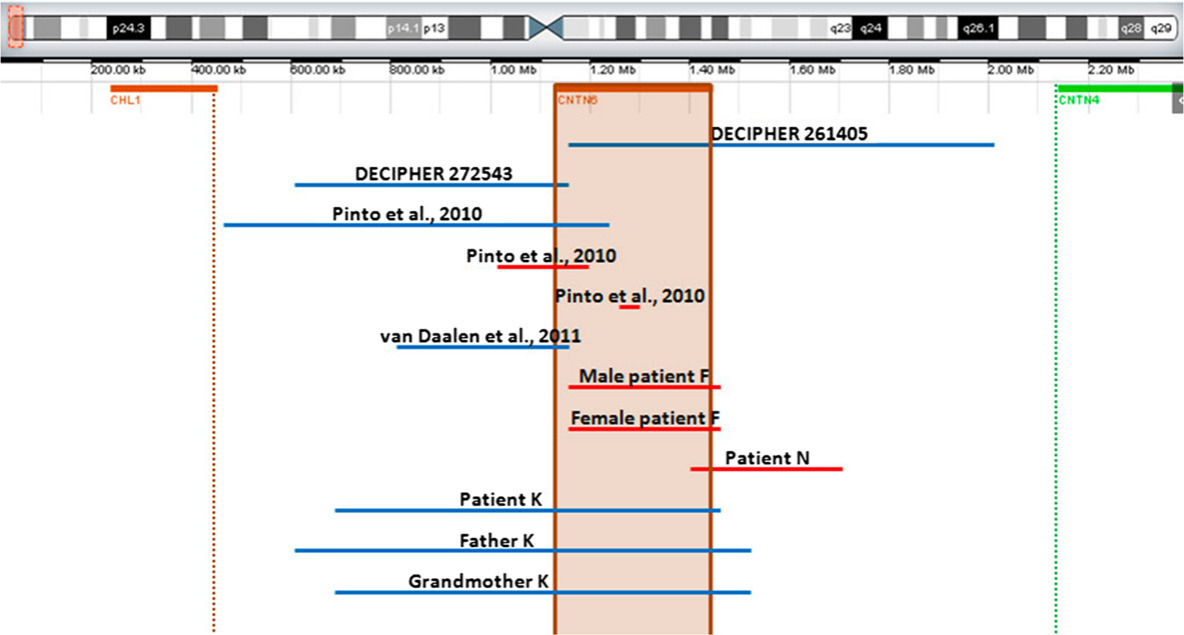
**A map of microdeletions and microduplications involving only** ***CNTN6*** **that have been published in DECIPHER and in the literature, as well as those that were discovered in our patients.** The microdeletions are shown in red, and the microduplications are colored blue.

No cases of microdeletion or microduplication involving only the *CNTN6* gene in association with ID have been described in the literature to date. However, unpublished data from six patients with these types of rearrangements can be found in the DECIPHER database. The sizes of these aberrations range from 0.14 to 1.86 Mb. A comprehensive map of *CNTN6* microdeletions and microduplications observed in patients with ASD and in some cases published exclusively in DECIPHER as well as those detected in our patients are shown in Figure 5. Unfortunately, clinical features were available for only two of the DECIPHER probands (Additional file 1: Table S1). Importantly, all of the patients assessed in this study and reported in the DECIPHER database, regardless of the type of the chromosomal aberration, had ID and psychomotor and growth retardation, except for patient N, who exhibited normal growth. In four of the six probands, low birth weight, microcephaly, and speech delay were registered, and three of the six had epicanthus, abnormality of the nose, wide nasal bridge, and abnormality of the feet (Additional file 1: Table S1). Abnormal eyebrows and prominent ears were common between our patient K and patient no. 272543 from DECIPHER, both of whom had dup3p26.3. These features were described only in patients with *CNTN6* duplications; therefore, they may be considered unique to this chromosomal aberration, in contrast with the deletion of *CNTN6*, according to the Golzio and Katsanis classification [17]. According to Preiksaitiene et al. [18], hydrocephalus (p = 0.023), downslanting palpebral fissures (p = 0.008), minor anomalies of the ear (p = 0.002), and micrognathia (p = 0.004) are significantly associated with pathogenic CNVs. Additionally, minor anomalies of the ear have been demonstrated to be predictors of pathogenic CNVs, increasing the risk by 3.5-fold.

It seems that all microdeletions and microduplication in our patients were inherited, although we could not perform examination of the parents of the affected sibs F. The microdeletion in patient N was inherited from an apparently healthy father. The microduplication in patient K was inherited from an apparently healthy father and grandmother. The microduplication in DECIPHER patient no. 272543 was also inherited from a normal father. The transmission of the microduplications and microdeletions of 3p26.3 from apparently healthy parents to their affected children may indicate that these CNVs have variable penetrance. According to the mode of inheritance, we also hypothesize that *CNTN6* may be an imprinted gene and suggest that it is expressed from the paternal allele and silenced on the maternal allele. Therefore, the father of patient K might inherit the inactive duplicated allele from his mother. Selective monoallelic gene expression resulted in normal health for both the father and grandmother. The allele became activated in the father’s germ cells, and this activated allele was inherited by his son. Therefore, his son received a double dose of the active gene and consequently, a double dose of the *CNTN6* protein, which may be pathogenic. The deletion of active *CNTN6* leads to a total absence of the protein and may result in disease, while the deletion of the imprinted allele has no deleterious effects unless there is a point mutation in *CNTN6* in the second allele. Another possibility may be related with the presence of several CNVs in genome and the modifying effect of one of them, which was first described by Girirajan et al. [19], who states that one CNV (for example, a 16p11.2 microdeletion) creates a susceptibility state, and additional CNVs are required for a more severe phenotype in an affected child. In family K along with microduplication 3p26.3 several other CNVs were detected, different in the patient, his father, and grandmother (Table 1). The affected boy has a polymorphic dup6p22.1p21.33 (112 kb) and an uncertain dup21q22.11 (164 kb). The latter has never been described in healthy controls, although its clinical significance is unclear as it contains only long intergenic non-protein coding RNA 159 gene (*LINC00159*). Therefore, following the Girirajan and colleagues hypothesis, there may be the modifying effect of other, even polymorphic CNVs’ effect on the development of disease. In family F, where both sibs are affected, the boy has the only microdeletion of *CNTN6*, when the girl has both exactly the same microdeletion of *CNTN6* and a polymorphic dup10q11.22, also observed in healthy individuals.

A terminal 2.9-Mb 3p26.3 microdeletion was followed for four generation in a family by Moghadasi et al. [20]. Thirteen members of this family were tested, and in seven of them, microdeletions were found. Only the sister of the index patient had the 3p26.3 microdeletion together with autism, speech delay, and learning difficulties. All other members with the microdeletion seemed to be healthy. All of the microdeletions in this family were maternally transmitted; thus, the authors also suggested that the distal 3p region may be imprinted. A female patient with a 3p26.3 microdeletion and ID also had a maternally inherited polymorphic 6q16.3 microduplication. Therefore, as an alternative hypothesis, the authors discussed the two-hit model of Girirajan et al. [20]. However, in the case of imprinting, a point mutation in the second active paternal allele may also have a deleterious effect and cause disease. A family with a maternally inherited 3p26.3 microduplication has also been described in the literature [21]. In addition to this rearrangement, the proband had a pathogenic 15q24 microdeletion, which may have explained his phenotype; however, his brother, who also possessed a 3p26.3 microduplication, was asymptomatic. This represents further support for the hypothesis of imprinting in the 3p26.3 region.

The comparison of the coordinates of the microdeletions and microduplication in our patients revealed that one coordinate (hg18, chr3:1,467,721) was exactly the same in three children from families F and K, indicating the possible reciprocal nature of these rearrangements due to non-allelic homologous recombination between segmental duplications (SDs). However, there is no evidence of SDs flanking this region.

The only gene encompassed by either a microdeletion or microduplication in our patients was *CNTN6*. *CNTN6* is a neuronal membrane protein that functions as a cell adhesion molecule and may contribute to the formation of axon connections in the developing nervous system. *CNTN6*, as well as other members of this protein family, have been identified as potential disease-causing genes in neurodevelopmental disorders. It has been suggested that these genes participate in pathways that are important for correct brain development [22]. In mice, they are thought to participate in embryonic development and postnatal brain maturation. *Cntn6* deficiency in mice causes profound motor coordination abnormalities and learning difficulties [23]. Additionally, a recent genome-wide association study has shown that this gene is associated with systemic lupus erythematosus [24]. The *CNTN6* haploinsufficiency index is 23.1%, which is close to the 0-10% that is considered to be indicative of haploinsufficiency (DECIPHER). This index value suggests that dose alterations of the *CNTN6* protein may affect the processes in which this protein is implicated. The insufficient amount hypothesis suggests that haploinsufficient genes are more sensitive to reductions than increases in dosage [25]. However, according to the gene balance hypothesis, the under- and overexpression of genes can lead to the same clinical phenotype, although the underlying molecular mechanisms differ.

## Conclusions

In conclusion, single-gene reciprocal microdeletions and microduplications are particularly significant for understanding the pathogenic effects of gene dosage alterations that are associated with CNVs. This study presented cases involving chromosomal aberrations that only encompassed the *CNTN6* gene. Our findings may help to elucidate the role and function of *CNTN6* in cognitive development.

## Methods

Two patients (the male patients F and K) were subjected to array CGH analysis because they were identified as having non-specific ID and mildly dysmorphic facial features during our previous investigation within the EU Seventh Framework Program project, “Improving Diagnoses of Mental Retardation in Children in Central Eastern Europe and Central Asia Through Genetic Characterisation and Bioinformatics/Statistics” (CHERISH) [26]. Because the male patient F had a single *CNTN6* gene deletion of 3p26.3 and the male patient K had a duplication in the same region and *CNTN6* is expressed in the brain, we considered these genetic aberrations to be pathogenic and continued analyzing these patients and their families. An additional array CGH analysis was performed for the intellectually disabled patient F’s sister and for the apparently healthy parents, aunt, cousin, and grandparents of patient K. Array CGH analysis of patient N was performed independently at the Department of Clinical Genetics, Copenhagen University Hospital, Rigshospitalet, Copenhagen, Denmark. IQ of patients was measured using the Wechsler IQ test.

Informed consent was obtained from the patients families or their representatives for publication of this study and any accompanying photographs.

### Ethical approval

The study was approved by the Bioethics Committee of the European Parliament.

### Conventional cytogenetic analysis

For all patients, conventional cytogenetic analysis was performed with GTG-banded metaphases of peripheral blood lymphocytes at a 400-500-band resolution.

### Array CGH

For the siblings from family F and patient K, array CGH analysis was performed using an Agilent 60K array at a resolution of approximately 41 kb (Human Genome CGH Microarray, Agilent Technologies, Santa Clara, CA, USA) according to the manufacturer’s recommendations. Data analysis was performed using Cytogenomics Software (v. 2.0.6.0) (Agilent Technologies, USA) and the publicly available Database of Genomic Variants [27] and the Database of Chromosomal Imbalance and Phenotype in Humans using Ensembl Resources [28].

For patient N, CNV analysis was performed using an Affymetrix CytoScan HD chromosome microarray platform (Affymetrix, Santa Clara, CA, USA) according to the manufacturer’s recommendations. Affymetrix CytoScan HD arrays provide 750,000 polymorphic (SNP, single nucleotide polymorphism) and 1,900,000 non-polymorphic (CNV) markers. The raw data were processed using Affymetrix Chromosome Analysis Suite software (ChAS), and the output data were interpreted with the UCSC Genome Browser (http://genome.ucsc.edu; GRCh37/hg19 assembly).

The functions of the *CNTN6* gene, which was located within the region of the genomic imbalances, were retrieved from the GeneCards database [29].

### Real-time quantitative PCR

Specific target sequences were selected for real-time quantitative PCR (qPCR) assay using Primer 3 software [30]. Two primer pairs were created that were specific for *CNTN6*. To confirm the integrity of the neighboring *CHL1* and *CNTN4* genes, one primer pair for each of these genes was also created.

For patient N, qPCR was performed using Power SYBR Green PCR technology (Applied Biosystems, Foster City, CA), and the data were normalized against *GAPDH* using the comparative ΔCT-method. All primers and conditions are available upon request.

## Additional file

Additional file 1: Table S1. Phenotypic features of patients with 3p deletion syndrome and del/dup of *CNTN6.*

## Abbreviations

Array CGH: Array comparative genomic hybridization
ASD: Autism spectrum disorder
ChAS: Chromosome Analysis Suite software
*CHL1*: Cell adhesion molecule L1-like
CNS: Central nervous system
*CNTN4*: Contactin 4
*CNTN6*: Contactin 6
CNV: Copy number variation
*CRBN*: Cereblon
DECIPHER: Database of Chromosomal Imbalance and Phenotype in Humans Using Ensembl Resources
EU: European Union
*GAPDH*: Glyceraldehyde-3-phosphate dehydrogenase
ID: Intellectual disability
IQ: Intellectual quotient
MRI: Magnetic resonance imaging
OMIM: Online Mendelian Inheritance in Man
PCR: Polymerase chain reaction
SNP: Single nucleotide polymorphism
