# Supplementary material for: Single gene microdeletions and microduplication of 3p26.3 in three unrelated families: CNTN6 as a new candidate gene for intellectual disability
Source: Mol Cytogenet. 2014 Dec 31;7:97. doi: 10.1186/s13039-014-0097-0 (PMC4299808; doi:10.1186/s13039-014-0097-0)
Supplement: Additional file 1: Table S1. — Phenotypic features of patients with 3p deletion syndrome and del/dup of CNTN6. [file 13039_2014_97_MOESM1_ESM.docx]

**Supplementary Table 1** Phenotypic features of patients with 3p deletion syndrome and del/dup of *CNTN6*

| Phenotype | Deletion involving *CNTN6* | | | | Duplication involving *CNTN6* | | |
| --- | --- | --- | --- | --- | --- | --- | --- |
|  | 3p deletion  syndrome | Male patient F  arr[hg18]3p26.3  (1,172,623-1,467,721)×1 | Female patient F  arr[hg18]3p26.3  (1,172,623-1,467,721)×1 | Female patient N hg[18]3p26.3  (1,403,385 -1,675,322)×1 pat | Male patient K  arr[hg18]3p26.3  (701,645-1,467,721)×3 pat | DECIPHER, patient no. 261405  hg[18]3p26.3  (1,172,657 -2,062,244)×3 | DECIPHER, patient no. 272543  hg[18]3p26.3  (641,287 –  1,172,653)×3 pat |
| Low birth weight | + | + | + |  |  |  | + |
| Feeding difficulties during infancy |  |  | + |  |  |  | + |
| Psychomotor and growth retardation | + | + | + | +* | + | + | + |
| Microcephaly | + | + | + |  | + |  | + |
| Trigonocephaly | + |  |  |  |  |  |  |
| Tower skull |  | + |  |  |  |  |  |
| Dolichocephalic skull |  |  |  |  | + |  |  |
| Moderate intracranial hypertension |  | + |  |  | + |  |  |
| Long face |  |  | + |  |  |  |  |
| Abnormality of the forehead |  | + |  |  |  |  |  |
| Abnormality of the eyebrows |  |  |  |  | + |  | + |
| Downslanting palpebral fissures | + | + |  |  |  |  |  |
| Upslanting palpebral fissures |  |  |  |  | + |  |  |
| Ptosis | + |  |  |  |  |  |  |
| Telecanthus | + |  |  |  |  |  |  |
| Epicanthus |  | + | + |  | + |  |  |
| Convergent strabismus |  |  |  |  | + |  |  |
| Hyperopia, amblyopia |  |  | + |  |  |  |  |
| Abnormality of the nose |  | + |  |  | + |  | + |
| Wide nasal bridge |  | + | + |  | + |  |  |
| Prominent ears |  |  |  |  | + |  | + |
| Low-set ears |  | + |  |  | + |  |  |
| Small ears |  |  | + |  |  |  |  |
| Preauricular pits | + |  |  |  |  |  |  |
| Short philtrum |  |  | + |  | + |  |  |
| Long philtrum |  |  |  |  |  |  | + |
| Micrognathia | + |  |  |  | + |  |  |
| Macrostomia |  |  |  |  | + |  |  |
| Cleft palate | + |  |  |  |  |  |  |
| High palate |  | + |  |  | + |  |  |
| Peg-shaped lateral incisors, crowding |  | + | + |  |  |  |  |
| Scoliosis |  | + | + |  | + |  |  |
| Abnormality of the feet (flatfoot, planovalgus deformity) |  | + | + |  |  |  | + |
| 2^nd^-3^rd^ toe syndactyly |  |  |  |  |  |  | + |
| Bilateral sandal gap |  | + | + |  |  |  |  |
| Bilateral V finger clinodactyly |  | + | + |  |  |  |  |
| Postaxial polydactyly | + |  |  |  |  |  |  |
| X-shaped legs |  |  | + |  |  |  |  |
| Hypotonia | + |  |  | + | + |  |  |
| Hypermobility of the small and large joints |  |  |  | + | + |  |  |
| Renal anomalies | + |  | + |  |  |  |  |
| Congenital heart defects | + |  |  |  | + |  |  |
| Sacral dimple | + |  |  |  |  |  |  |
| Gastrointestinal anomalies | + |  |  |  |  |  | + |
| Cryptorchidism |  |  |  |  | + |  |  |
| Testicular hypoplasia |  | + |  |  |  |  |  |
| Varicocele |  | + |  |  |  |  |  |
| Hypospadias |  |  |  |  | + |  |  |
| Seizures |  | + |  |  |  | + |  |
| Apraxia |  |  |  |  | + |  |  |
| Hyperkinesis |  |  |  |  | + |  |  |
| Reduced bone age |  | + |  |  |  |  |  |
| Capillary hemangiomas |  |  |  |  |  | + |  |
| Non-malignant pigmented nevus |  | + |  |  |  |  |  |
| Hypertrichosis |  | + |  |  |  |  |  |
| Coarse hair |  |  |  |  | + |  |  |
| Thyroid gland hypoplasia |  | + | + |  |  |  |  |
| Intellectual disability | + | + | + | + | + | + | + |
| Speech delay |  | + |  | + | + |  | + |
| Language delay |  |  | + |  |  |  |  |
| Exhaustible attention |  | + |  |  |  |  |  |
| Reduced memory |  |  | + |  |  |  |  |
| ADHD |  |  |  |  | + |  |  |
| Atypical autism |  |  |  | + |  |  |  |

Footnote. * - The characteristics of the development of the patients are discussed in the manuscript. Symptoms found in all patients with a microdeletion or microduplication are shown in gray; those found in three to four patients out of six with a microdeletion or microduplication are colored blue; those found only in patients with a microdeletion are colored green; and those identified only in patients with a microduplication are colored pink.
